# Biomarkers for the toxicity of sublethal concentrations of triclosan to the early life stages of carps

**DOI:** 10.1038/s41598-020-73042-y

**Published:** 2020-10-14

**Authors:** Owias Iqbal Dar, Sunil Sharma, Kirpal Singh, Anket Sharma, Renu Bhardwaj, Arvinder Kaur

**Affiliations:** 1 Aquatic Toxicology Lab, Department of Zoology, Guru Nanak Dev University, Amritsar, Punjab 143005 India; 2 Plant Stress Physiology Lab, Department of Botanical and Environmental Sciences, Guru Nanak Dev University, Amritsar, Punjab 143005 India; 3 State Key Laboratory of Subtropical Silviculture, Zhejiang A & F University, Hangzhou, 311300 China

**Keywords:** Zoology, Environmental sciences

## Abstract

Accumulation, contents of protein, non-enzymatic antioxidant glutathione (GSH and GSSG), lipid peroxidation product (melondialdehyde-MDA) and organic acids (fumarate, succinate, malate and citrate), and activities of neurological (acetylcholinesterase-AChE), detoxification (glutathione S-transferase-GST) and metabolic (lactate dehydrogenase-LDH, aspartate transaminase-AST and alanine transaminase-ALT) enzymes were recorded in the hatchlings of *Cyprinus carpio*, *Ctenopharyngodon idella*, *Labeo rohita* and *Cirrhinus mrigala* after 7 and 14 days exposure and 10 days post exposure (recovery period) to sublethal concentrations (0.005, 0.01, 0.02 and 0.05 mg/L) of triclosan, a highly toxic and persistent biocide used in personal care products. Accumulation was maximum between 7–14 days at 0.01 mg/L for *C. carpio* and *L. rohita* but at 0.005 mg/L for *C. idella* and *C. mrigala*. No triclosan was observed at 0.005 mg/L in *C. carpio* and *C. mrigala* after recovery. Significant decline in protein, glutathione and acetylcholinesterase but increase in glutathione S-transferase, lactate dehydrogenase, aspartate transaminase, alanine transaminase, melondialdehyde and organic acids over control during exposure continued till the end of recovery period. Integrated biomarker response (IBR) analysis depicted higher star plot area for glutathione and glutathione S-transferase during initial 7 days of exposure, thereafter, during 7–14 days of exposure and the recovery period, higher star plot area was observed for acetylcholinesterase, aspartate transaminase, alanine transaminase and organic acids. Higher star plot area was observed for protein in all the species throughout the study. The study shows that *L. rohita* is most sensitive and glutathione, acetylcholinesterase, aspartate transaminase and alanine transaminase are the biomarkers for the toxicity of sublethal concentrations of TCS.

## Introduction

Triclosan [TCS, 5-chloro-2-(2,4-dichlorophenoxy) phenol], a broad spectrum antibacterial agent has been placed on the list of ten most common pollutants of terrestrial and aquatic environments^1,2^. Its wide usage in personal care, plastic, textile and acrylic products and insufficient removal (58–90%) by wastewater treatment plants (WWTPs) are responsible for its continuous release into the aquatic bodies all over the world^3,4^. Its presence has been reported from surface waters, ground water, sediments of water bodies and tissues of several aquatic organisms like filamentous algae, freshwater snails, Atlantic bottlenose dolphins, Mediterranean mussel, amphibian larvae, including fish^5–8^. These studies also reported dose dependent increase in mortality and bioaccumulation of TCS in the organisms. Because of lipophilicity, TCS has the potential for bioaccumulation in the food web^9^ and is considered to be hazardous for the environment and life even at low concentration. Destructive impacts of TCS on aquatic organisms include, endocrine disruption^10^, neurotoxicity^11^, mitochondrial dysfunction^12^, oxidative stress due to increased production of various reactive oxygen species (ROS), alteration of the activity of antioxidative, detoxification and metabolic enzymes and impairment of immune system^13–17^.

For combating such degenerative effects of toxicants on the cellular components, all the organisms have a well-organized antioxidative defense system. However, when xenobiotics upset the equilibrium of ROS generation and activity of antioxidative and metabolic enzymes there is a further increase in the levels of ROS, peroxidation of lipids, degradation of proteins, inactivation of enzymes and damage to the genetic material in the stressed animals^18,19^. This may gradually lead to the death of the organisms if their antioxidant defense system fails to recover^20^. Initially, to adjust to such altered conditions, the tricarboxylic acid cycle (TCA) is geared up to maintain energy metabolism, macromolecule synthesis, redox balance and various signaling events^21,22^. Determination of the activity of antioxidative and metabolic enzymes along with the concentration of intermediates (organic acids) of the TCA cycle therefore provides clues for understanding the mechanisms that help the organisms to overcome a particular kind of stress^23^.

Aquatic bodies are the sinks for almost all kinds of pollutants on earth, therefore for toxicity tests, aquatic organisms, especially fish are preferred because of more sensitivity to stressors than the terrestrial organisms including mammals^24^. Fish has more physiological similarity with man, therefore the data obtained from such studies are directly applicable to various other organisms including man^25^. Higher susceptibility and sensitivity of early developmental stages of a fish to the toxins has made them most suitable indicators for environmental contamination. At the same time, top position of fish in the aquatic food web makes these stages more vulnerable to direct or indirect exposure to xenobiotics. Toxicity assays with the early developmental stages of fish have been therefore generally used for studying a wide range of endpoints^26^.

Globally, extended interactions of organisms with low non-fatal concentrations of chemicals in the environment are a major concern, and therefore regulatory emphasis is placed on long term exposures. Some reports on sublethal toxicity of TCS to fish are available^11,27–30^ but these deal with fathead minnows, zebrafish, rainbow trout and catfishes. However, EPA^31^ suggests that more comparative studies are needed as the super class pisces is extremely diverse and mere dependence on one or two species for drawing generalizations will not be a scientific approach. Hence in the present study, embryos of *Cyprinus carpio*, *Ctenopharyngodon idella*, *Labeo rohita* and *Cirrhinus mrigala* were selected as test models. The embryos were exposed for 14 days to four sublethal concentrations of TCS (0.005, 0.01, 0.02, 0.05 mg/L), within the range of observed levels of TCS in waters from several parts of the world^32^. The study was extended further for 10 days to observe prolongation of the post exposure stress effect. These carps are abundantly found in natural water bodies of India and are widely cultured and consumed by a large population. Apart from being commonly available, the eggs of these species are easy to handle during experimentation. The work holds importance as there is hardly any report on biochemical alterations in food fishes thriving such waters. Data for accumulation of TCS, contents of protein, glutathione (GSH and GSSG), melondialdehyde (MDA) and organic acids (fumarate, succinate, malate and citrate) and activity of acetylcholine esterase (AChE), glutathione S-transferase (GST), lactate dehydrogenase (LDH), aspartate transaminase (AST) and alanine transaminase (ALT) were subjected to Integrated biomarker response (IBR) for obtaining early biomarkers for the toxicity of sublethal concentrations of TCS after 7 and 14 days of exposure and 10 days of recovery period. Additionally, visible markers (hatching and developmental anomalies) of the toxicity were also recorded.

## Results

There was a species-specific variation in TCS accumulation and biochemical parameters of the selected fishes after the exposure as well as the recovery period. Species specific variation was also observed in percent mortality, percentage of abnormal hatchlings on exposure to TCS.

### TCS accumulation

**Figure 1:**
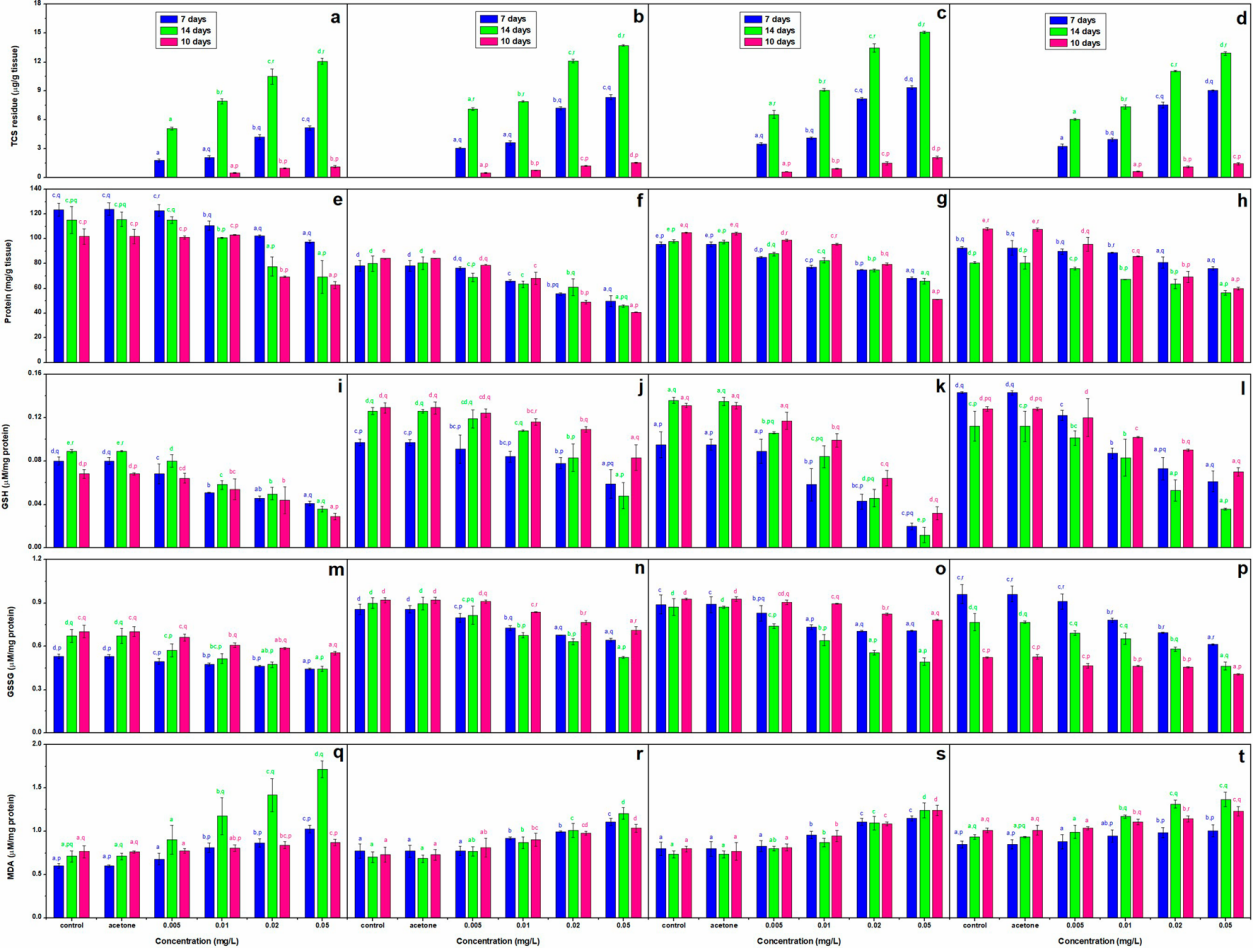
Content of TCS (**a–d**), protein (**e–h**), GSH (**i–l**), GSSG (**m–p**) and MDA (**q–t**) in the hatchlings of *C. carpio* (**a, e, i, m, q**), *C. idella* (**b, f, j, n, r**), *L. rohita* (**c, g, k, o, s**) and *C. mrigala* (**d, h, l, p, t**) after 7 and 14 days exposure and 10 days of recovery period. Bars with letters a-e are significantly (*p* < 0.05) different among treatments and with p–r are significantly (*p* < 0.05) different with respect to duration of exposure, values are mean ± SD, n = 4.

There was a concentration dependent increase (*p* < 0.05) in accumulation of TCS in the hatchlings during the exposure, and it continued during the post exposure period. About 10% of triclosan in water got accumulated in the hatchlings during 14 days of exposure, maximum increase over 7 days values in TCS accumulation was observed at 0.01 mg/L for *C. carpio* (+ 276.92%) and *L. rohita* (+ 121.03%) but at 0.005 mg/L for *C. idella* (+ 132.32%) and *C. mrigala* (+ 85.72%). At the end of the recovery period, content of TCS declined over its respective 7 days values (*p* < 0.05) in all the species (Fig. 1a–d). Complete elimination of TCS was observed only in *C. carpio* and *C. mrigala* at 0.005 mg/L, while at 0.01, 0.02 and 0.05 mg/L, the decline ranged between 76–78% in *C. carpio*, 79–83% in *C. idella*, 77–81% in *L. rohita* and 83–85% in *C. mrigala*.

### Biochemical parameters

TCS caused a concentration dependent decline (*p* < 0.05) in protein, GSH, GSSG and AChE but an increase (*p* < 0.05) in GST (except for a continuous decline till the end of recovery period at 0.05 mg/L in all but *C. mrigala* but till 14 days of exposure at 0.02 mg/L in *C. idella* and *L. rohita* only), LDH (except for a non-significant increase on the 10th day of recovery period in *C. carpio*), AST, ALT and MDA on all the durations (Fig. 1e–t, 2a–t). However, GSH/GSSG ratio exhibited a decline (*p* < 0.05) till the 14th day of exposure but an increase (*p* < 0.05) on the 10th day of recovery period.

### Protein content

TCS induced changes in protein content have been presented in Fig. 1e–h. After the initial 7 days of exposure, maximum and minimum decline over control in protein content with the concentration of TCS was observed in *C. idella* (36.59%) and *C. mrigala* (17.93%), respectively. Between 7 to 14 days, there was 1.5 times higher decline over control in the content of protein in *C. idella*, *L. rohita* and *C. mrigala*, but it was 2 times higher in *C. carpio*. During the post exposure period of 10 days, protein content declined over control further in *C. idella* (51.63%), *L. rohita* (51.31%) and *C. mrigala* (44.56%) only.

### GSH

A continuous decline in GSH till 14 days of exposure was followed by lesser decline after 10 days of recovery period in all the species (Fig. 1i–l). After 14 days, decline over control in *L. rohita*, *C. mrigala*, *C. idella* and *C. carpio* was 91.18%, 67.86%, 61.90% and 59.55%, respectively. On the other hand, after recovery, the decline over control was 75.57% in *L. rohita*, 57.35% in *C. carpio*, 45.32% in *C. mrigala* and 35.66% in *C. idella*.

### GSSG

A continuous decline in GSSG till the end of exposure showed some improvement after the recovery period in all the fish except for *C. carpio* (Fig. 1m–p). After 14 days, decline over control in the content of GSSG was 1.5–2.0 times more than the 7 days values with maximum and minimum decline over control in *L. rohita* (43.40%) and *C. carpio* (33.88%). On the other hand, maximum and minimum decline over control after recovery was noticed in *C. idella* (22.63%) and *L. rohita* (16.64%).

### MDA

Figure 1q–t highlight TCS induced changes in GSH. After 14 days of exposure, content of MDA exhibited 1.5–2.0 times more increase over control compared to 7 days in all the fish. On this day, it increased by 141.35% in *C. carpio*, 71.65% in *C. idella*, 69.35% in *L. rohita* and 46.52% in *C. mrigala* at 0.05 mg/L compared to control. After recovery, the increase over control was less compared to both the durations of exposure in *C. mrigala*, *C. carpio* and *L. rohita*, while in *C. idella*, a further increase was observed at 0.005 and 0.01 mg/L. On this day, MDA content was maximum in *L. rohita* (56.16%) and minimum in *C. carpio* (13.84%).

### AChE

**Figure 2:**
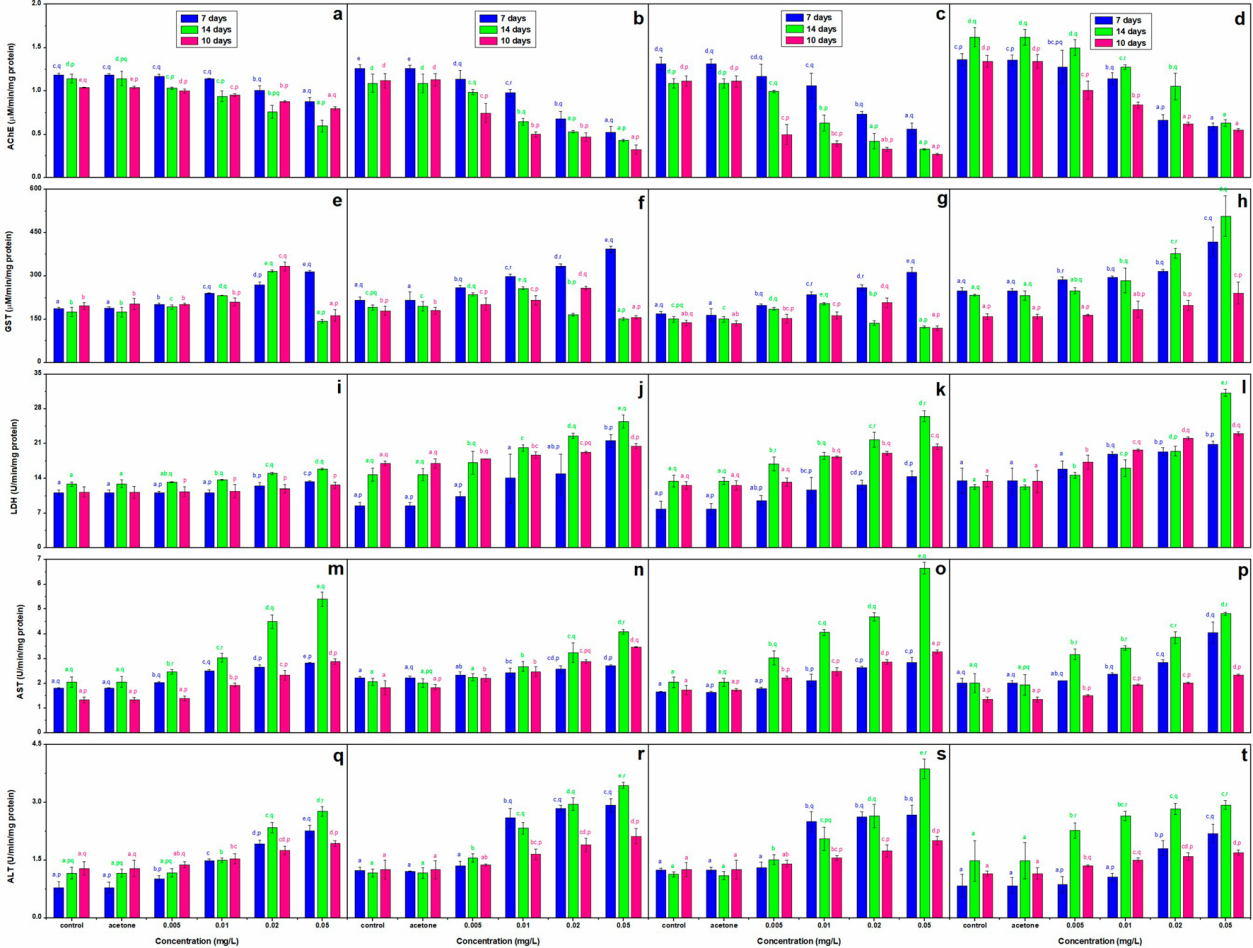
Effect of TCS on the activity of AChE (**a–d**), GST (**e–h**), LDH (**i–l)**, AST (**m–p**), and ALT (**q–t)** in *C. Carpio* (**a, e, i, m, q**), *C. idella* (**b, f, j, n, r**), *L. rohita* (**c, g, k, o, s**) and *C. mrigala* (**d, h, l, p, t)** after 7 and 14 days exposure and 10 days of recovery period. Bars with letters a-e are significantly (*p* < 0.05) different among treatments and with p–r are significantly (*p* < 0.05) different with respect to duration of exposure, values are mean ± SD, n = 4.

Figure 2a–d show that after 14 days of exposure, decline in AChE activity compared to control was highest (61.07%) in *C. mrigala* and lowest (47.86%) in *C. carpio* but after 10 days of recovery period, there was improvement in enzyme activity as the decline over control was 59.27 and 23.17%, respectively. On the other hand, AChE showed a continuous decline over control till the end of recovery period in *C. idella* and *L. rohita* (76.08 and 71.16%, respectively).

### GST

Till 7 days of exposure, GST activity increased over control in all the fish, it was maximum in *L. rohita* (83.94%) and minimum in *C. carpio* (67.86%), thereafter a species specific variation was observed in enzyme activity (Fig. 2e–h). It showed continuous increase over control till the end of recovery period in *C. mrigala,* the increase over control was 16.79% after 14 days and 51.16% after 10 days of recovery period. On the other hand, continuous decline over control was observed in GST activity of *C. carpio*, *L. rohita*, and *C. idella* at 0.05 mg/L (16.82%, 13.79% and 12.80%, respectively). In *C. idella* and *L. rohita,* enzyme activity declined over control even at 0.02 mg/L after 14 days of exposure but then increased compared to control after the recovery period.

### LDH

LDH activity of the exposed fish was higher than control after exposure as well as recovery except for a slight decline at 0.005 mg/L after 7 days in *C. carpio* (Fig. 2i–l). However, after recovery, the increase over control was 1/2 to 1/3 times less than both the durations of exposure. After 7 days, highest increase over control was 153.41% in *C. idella*, 82.43% in *L. rohita*, 54.70% in *C. mrigala* and 19.47% in *C. carpio*. Activity increased further till 14th day in *L. rohita* (97.63%) and *C. mrigala* (155.23%) only. After recovery, LDH activity came down but still it was 71.95, 65.52, 21.09 and 12.68% more than control in *C. mrigala*, *L. rohita*, *C. idella* and *C. carpio*, respectively.

### AST

Figure 2m–p highlight that by the 14^th^ day of exposure, the concentration dependent increase over control in AST activity was 2–3 times more than the 7 days values. After recovery it came down but remained more than 7 days values in all the species. Enzyme activity after recovery was 116.27%, 89.56%, 88.89% and 73.72% more than control in *C. carpio*, *L. rohita*, *C. idella* and *C. mrigala*, respectively.

### ALT

Figure 2q–t show a continuous increase over control in ALT activity till 14 days at all the concentrations in *L. rohita* and *C. idella*. However, the increase over control on 14th day was less than 7 days in *C. carpio* (at all the concentrations) and *C. mrigala* (at 0.02 and 0.05 mg/L). After recovery, ALT activity came down, but it was still 68.79%, 59.67%, 50.39% and 47.19% more than control in *C. idella*, *L. rohita*, *C. carpio* and *C. mrigala*, respectively.

### Organic acids

**Figure 3:**
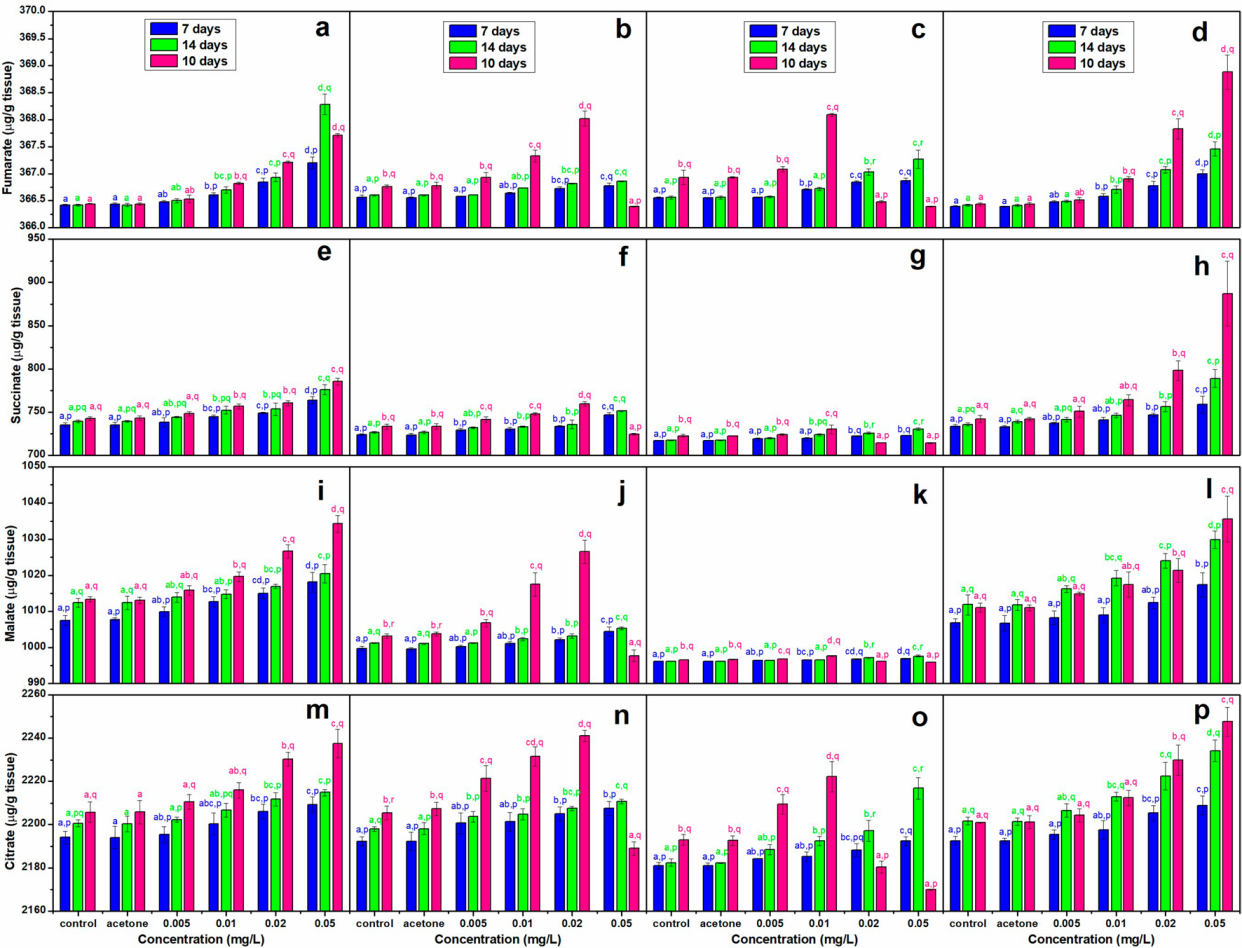
Effect of TCS on the content of fumarate (**a–d**), succinate (**e–h**), malate (**i–l)** and citrate (**m–p**) in *C. Carpio* (**a, e, i, m**), *C. idella* (**b, f, j, n**), L. rohita (**c, g, k, o**) and C. mrigala (**d, h, l, p)** after 7 and 14 days exposure and 10 days of recovery period. Bars with letters a–e are significantly (*p* < 0.05) different among treatments and with p-r are significantly (*p* < 0.05) different with respect to duration of exposure, values are mean ± SD, n = 4.

A significant (*p* < 0.05) concentration and duration dependent increase (except for a decline at 0.02 mg/L in *L. rohita* and at 0.05 mg/L in *C. idella* and *L. rohita,* after 10 days of recovery period) was observed in the contents of the selected organic acids in all the species(Fig. 3). Maximum increase was observed in succinate throughout the experiment in all the species with highest value in *C. carpio* (+ 3.92%), after 7 days of exposure but in *C. mrigala,* after 14 days of exposure and 10 days of recovery period (+ 7.21 and + 19.56%, respectively). On the other hand, fumarate exhibited minimum increase in the TCS exposed hatchlings (except for malate in *L. rohita* at the end of recovery period, at all the concentrations) throughout the experiment. Of the four fish, fumarate level was highest in *C. carpio* (+ 0.21%) after 7 days of exposure but in *C. mrigala,* after 14 days of exposure (+ 0.29%) as well as 10 days of recovery period (+ 0.67%), while its lowest level was observed in *C. idella* after 7th, 14th and 10th day (+ 0.06, + 0.07 and + 0.34%, respectively).

### Integrated biomarker response (IBR)

**Figure 4:**
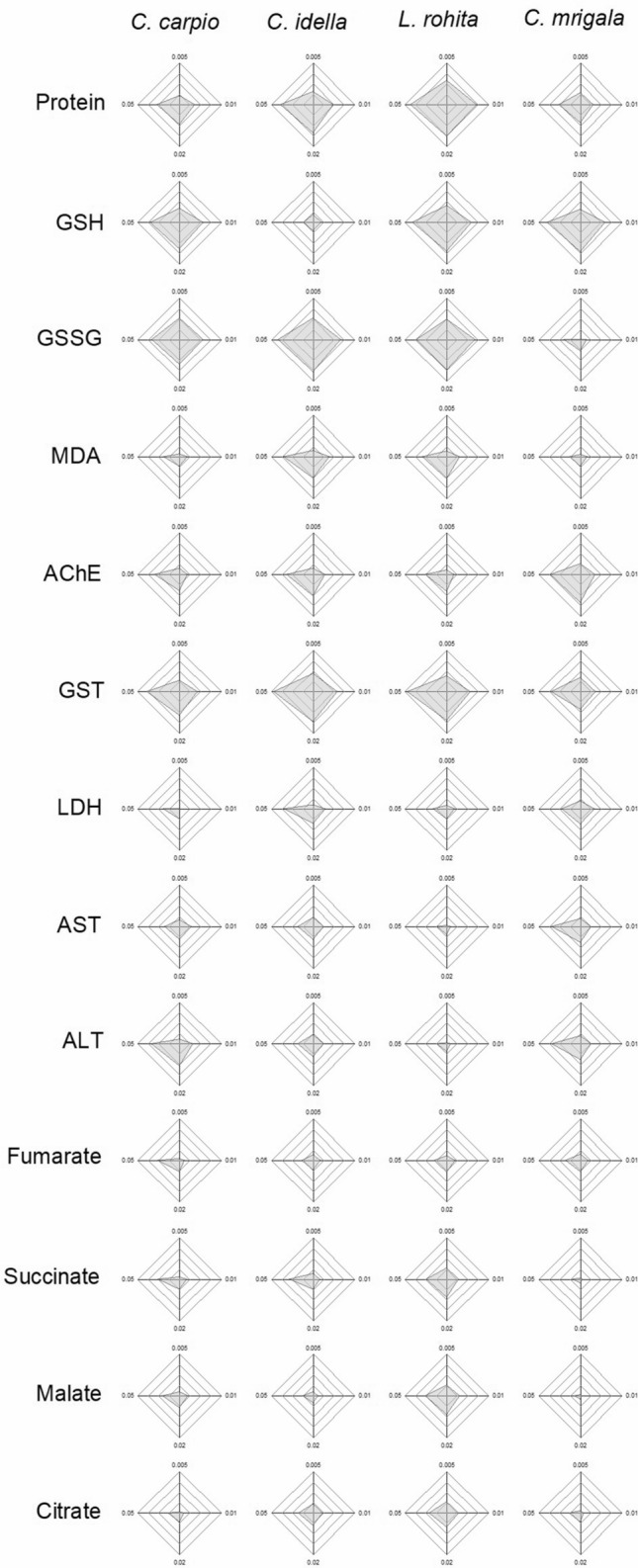
IBR star plots for multi-biomarker response in different fishes after 7 days of exposure to different concentrations of TCS.

**Figure 5:**
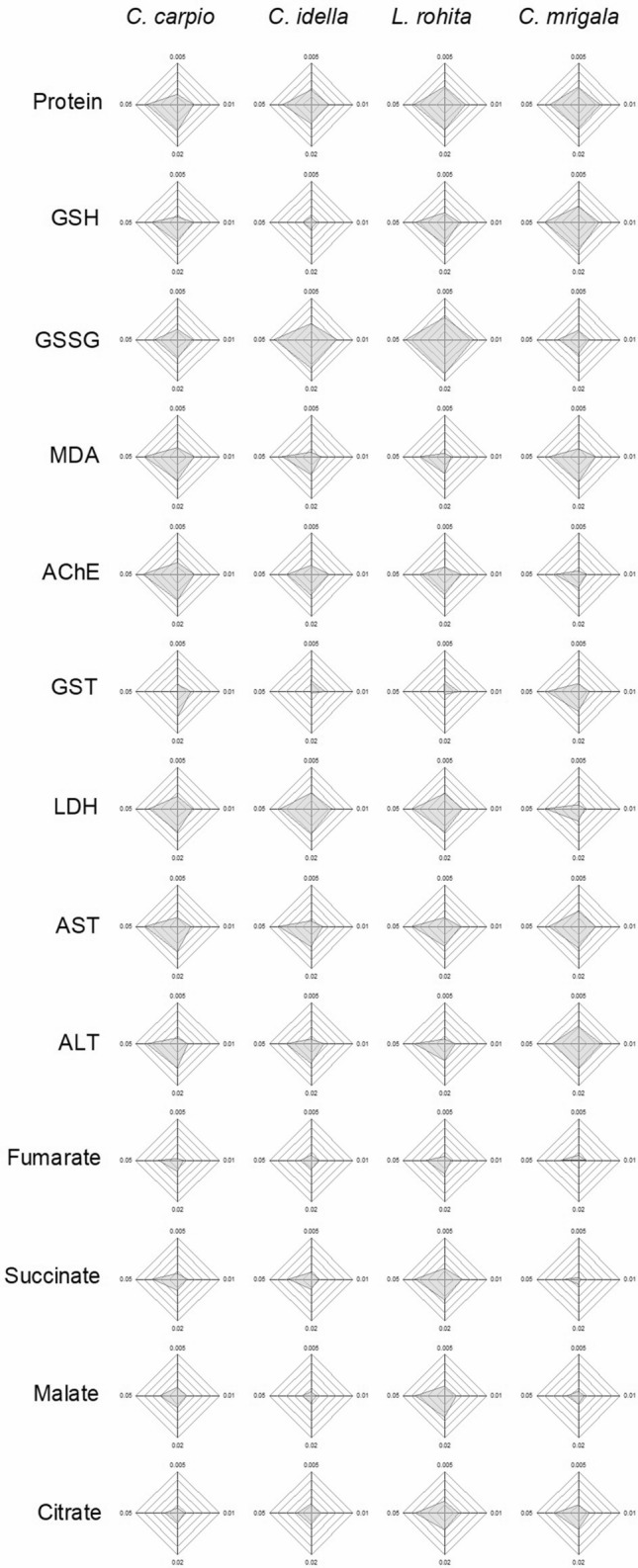
IBR star plots for multi-biomarker response in different fishes after 14 days of exposure to different concentrations of TCS.

**Figure 6:**
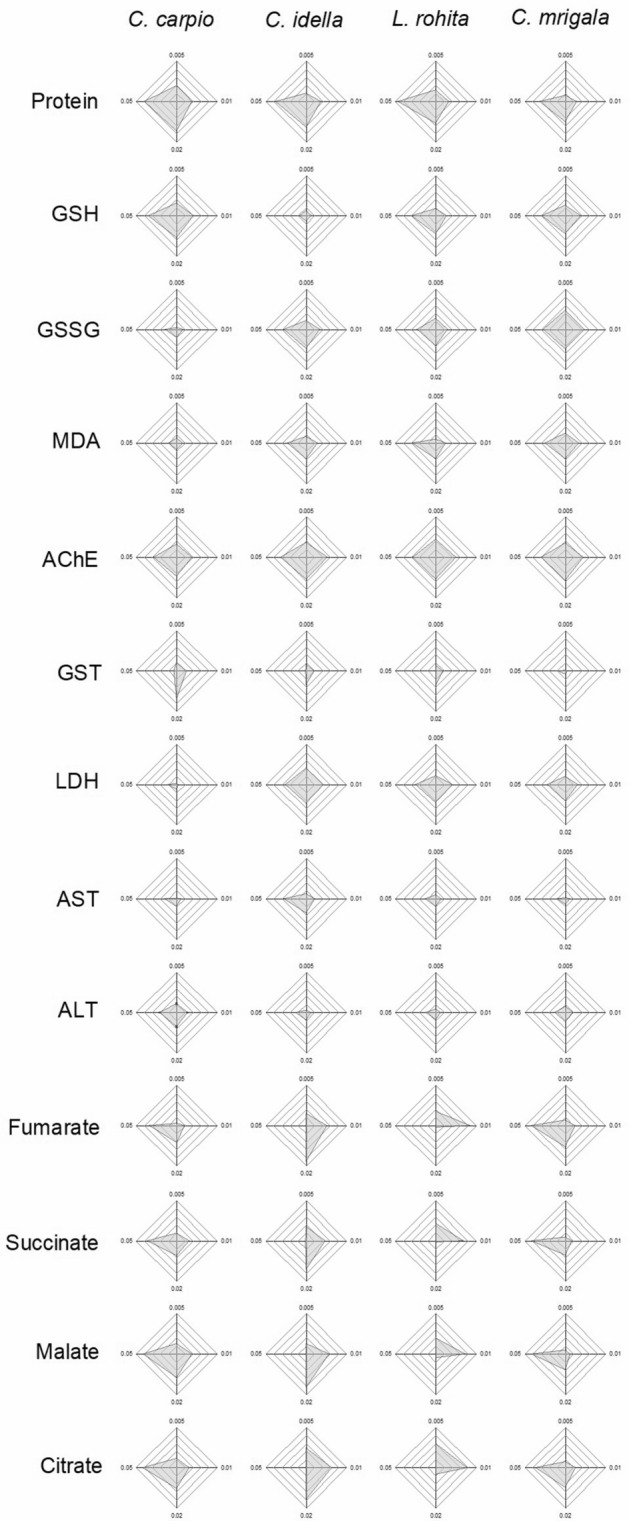
IBR star plots for multi-biomarker response in different fishes after 10 days of the recovery period.

IBR (Table 1, Fig. 4–6) showed a differential biomarker response in different species. When five biomarkers with higher star plot area were selected, it was observed that during the initial 7 days of exposure, oxidative stress predominated in the hatchlings as the area of the plots for GSH, GSSG and GST was higher than other factors for all the fish. Gradually, metabolic stress increased during 7–14 days as area of MDA, AChE, AST and ALT also increased. During the recovery period also, area of AChE remained higher than other factors for all the fish. At the same time, area of organic acids also increased during this period. Protein was observed to be one of the top five scorers for all the species throughout the study.

**Table 1:** Integrated biomarker response (IBR) values of different biomarkers in each fish after 7 and 14 days of exposure and 10 days of the recovery period. Values are sum of corresponding IBRs ($\mathop \sum \limits_{{{\text{i}} = 1}}^{4} {\text{A}}_{{\text{i}}}$).

Biomarker	*C. Carpio*			*C. idella*			*L. rohita*			*C. mrigala*		
	7 days	14 days	10 days	7 days	14 days	10 days	7 days	14 days	10 days	7 days	14 days	10 days
Protein	4.99	12.46	16.35	9.99	11.30	11.70	17.00	16.01	12.93	4.80	15.00	7.52
GSH	10.02	7.60	11.90	1.38	1.20	0.94	12.28	10.03	6.81	11.15	17.31	8.45
GSSG	10.94	7.78	1.37	15.44	20.03	7.74	13.74	27.00	6.42	1.07	5.25	12.19
MDA	1.34	11.50	1.20	5.76	6.25	5.24	4.83	4.46	4.99	0.92	11.17	6.77
AChE	3.32	13.94	9.58	4.87	8.98	13.13	2.87	7.65	13.24	7.90	3.87	11.86
GST	8.10	3.27	3.49	13.98	1.32	1.41	13.06	1.45	1.04	6.59	8.51	0.28
LDH	0.77	11.04	0.48	3.55	15.10	10.31	1.34	13.59	7.68	3.48	4.65	5.74
AST	2.44	11.20	1.02	2.63	8.14	4.67	0.56	9.85	1.32	4.33	12.45	0.68
ALT	5.02	8.87	5.00	5.17	6.51	1.16	4.48	6.41	1.13	1.72	14.76	2.12
Fumarate	1.59	2.30	5.19	1.24	1.96	7.60	1.57	3.14	4.19	1.79	0.95	8.90
Succinate	1.67	4.18	7.12	2.19	3.57	6.33	4.31	10.53	3.93	0.20	0.77	5.55
Malate	1.85	3.79	12.62	0.71	1.12	7.68	4.65	9.13	4.75	0.15	1.56	5.87
Citrate	0.73	2.19	10.69	2.45	3.54	10.21	3.45	8.92	7.33	0.74	5.97	8.02

### Hatching, mortality and abnormalities

**Figure 7:**
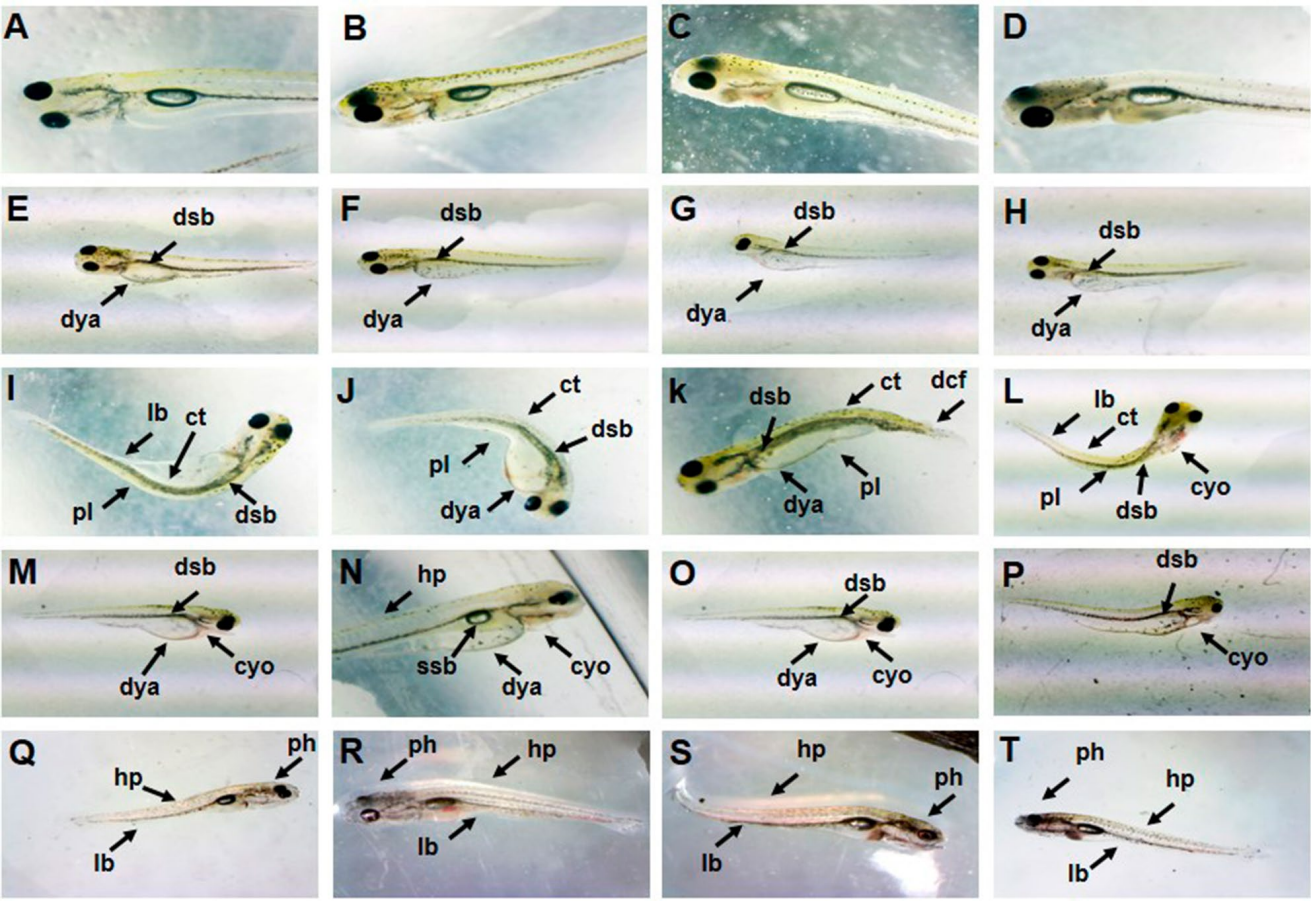
Normal larvae (**A–D**) and abnormalities induced by sublethal concentrations of TCS in the hatchlings (**E–T**) of *C. carpio* (**A, E, I, M, Q**), *C. idella* (**B, F, J, N, R**), *L. rohita* (**C, G, K, O, S**) and *C. mrigala* (**D, H, L, P, T**). **dsb**-deflated swim bladder, **dya**-delayed yolk absorption, **pl**-paralytic larvae, **ct**-curved tail, **dcf**-deformed caudal fin, **cyo**-cardiac and yolksac oedema, **ssb**-small swim bladder, **hp**-hypopigmentation, **ph**-pointed head and **lb**-lean body.

Figure 7 shows normal larvae (A–D) and TCS induced abnormalities (E–T) in the hatchlings of four carps. It was observed that the selected concentrations of TCS caused no delay/decline in hatching but induced developmental abnormalities in all the species. At 0.005 and 0.01 mg/L, around 1–2% hatchlings showed delayed yolk absorption (Fig. 7E–H), deflated swim bladder (Fig. 7E–H), curved tail (Fig. 7I–L), cardiac and yolk-sac oedema (Fig. 7M–P) and hypopigmentation (Fig. 7Q–T) while at 0.02 and 0.05 mg/L, this frequency increased to 3–8% in *C. carpio* and *C. mrigala* but to 10–15% in *C. idella* and *L. rohita*. Till four days of exposure, the hatchlings swam with a slow speed but 5^th^ day onwards, 15–20% hatchlings of *C. carpio* and 30–40% hatchlings of the other species became paralytic at 0.02 and 0.05 mg/L TCS and showed only wriggling movement even after prodding with a rod (Fig. 7I–L). Between 10–14 days of exposure, paralytic hatchlings (all the species) were observed even at 0.01 (15–20%) and 0.005 (3–5%) mg/L TCS. The paralyzed larvae ate very less, became very thin (lean body) and had a pointed head by the end of experiment (Fig. 7Q–T). Till 7 days, no mortality was observed in control, solvent control, 0.005 and 0.01 mg/L but by 14^th^ day, it increased to 2–4% at 0.005 and 0.01 mg/L. On the other hand, at 0.02 and 0.05 mg/L, there was a marked increase in mortality between 7 (2–3%) to 14 (15–20%) days and it persisted at 5–10% during the recovery period. Highest percentage of abnormalities and mortality was observed in *L. rohita*.

## Discussion

The observed concentration and duration dependent increase in the accumulation of TCS is probably due to its high log Kow value (4.8). There are generally two routes of absorption of a pollutant from the aquatic environment i.e. through direct contact and through contaminated food. In the present experiment whole body surface of hatchlings was in direct contact of the contaminated water because it was devoid of scales. The scales develop 20 days post hatching^33,34^ and protect the skin from predators, parasites and pollutants in the surrounding environment. This gets support from Escarrone et al.^35^, who observed that accumulation of TCS in different tissues of *Poecilia vivipara* reached a steady state after one week of exposure in most of the tissues except for gill (having large surface area in direct contact with the pollutant) and gonad (having large lipid content). Minimum content of TCS in *C. carpio* compared to other three fishes seems likely due to the observed longer hatching time (57 h) than the other three fishes (18–22 h). This might have delayed absorption through both, contact and feeding in the hatchlings. External feeding in hatchlings starts 24–36 h post hatching^36^. However, during the recovery period of 10 days, 76–100% decline in the level of TCS shows that hatchlings of all the species could metabolize and excrete TCS. Species specific variation in the TCS content of the fishes of this study is corroborated by Palenske et al. ^7^.

Lesser protein content of the TCS exposed hatchlings is an indication of protein catabolism in them for increased energy demand for repairing the wear and tear. Proteins not only play a significantly important role in almost all structures and functions of living organisms but are also used for energy generation and maintenance of homeostasis under normal and stressed conditions^37^. Decline in protein has also been related to weakening/damage to the synthesizing machinery of fish under stress in previous studies^38–40^. The cells generally maintain essential functions by promoting or inhibiting synthesis or degradation of proteins^41^, however, under stress, protein degradation becomes a key process for production of ATP, liberation of amino acids and other substances for gluconeogenesis, synthesis of antioxidants and detoxification enzymes and to meet the energy demands^42–44^. TCS is known to induce ROS generation in organisms^13,45^ which cause carbonyl protein formation and as a result of this, there is change in the conformation of proteins, increase in photolytic susceptibility and decline in enzyme activity^38,46^. Less intake of feed by the exposed hatchlings seems to be an additional factor for further decline in protein content during the post exposure period. Maximum decline in *L. rohita* and *C. idella* than the other two species was parallel to the higher accumulation of TCS in these fish.

Continuous decline in the content of glutathione (GSH and GSSG) in the present study indicates that TCS disturbed ROS homeostasis and led to oxidative stress and lipid peroxidation in all the species. Oxidative stress of TCS was also visible as simultaneous increase in MDA content in the exposed hatchlings of all the species in our study. An effort of the hatchlings to get rid of TCS or its metabolic products or ROS may have resulted in a continuous concentration dependent decline in the contents of GSH and GSSG till the end of recovery period as suggested by Monteiro et al.^47^ and Lu^48^. Increase in GST activity (responsible for conjugation reactions) also corresponded with the decrease of GSH after 7 days of exposure to TCS in all the fish. However, after 14 days of exposure, the stress seems to have overpowered the defense mechanism as there was a decline in GST as well as GSH and GSSG in *C. carpio*, *C. idella* and *L. rohita*. In *C. mrigala,* GST increased but GSH and GSSG declined on all the durations.

GSH binds with ROS to reduce their adverse effects^49^ therefore, higher levels of GSH and high GSH/GSSG ratio are mandatory to overcome stress and maintain normal functioning of cells^50,51^. Persistent decline in the ratio of GSH/GSSG in the exposed hatchlings compared to control till end of recovery period hints towards prolonged oxidative stress of the selected sublethal concentrations of TCS in all the species. Oxidative stress of TCS was visible as concentration dependent increase in MDA level till the end of the recovery period. Higher MDA level is an indicator of lipid and cellular damage in stressed animals^52,53^.

Continuous decline in AChE activity after both the durations of exposure as well as 10th day of recovery period is an indicator of the neurotoxic effect of the selected sublethal concentrations of TCS. Studies of Arias-Cavieres et al.^54^ and Szychowski et al.^55^ that TCS induced apoptosis as well as inhibition of N-methyl-D-asparagic acid receptor and Ca^2+^ transport in neocortical or hippocampal neurons clearly point towards its neurotoxic potential. Alteration of enzyme synthesis pathways and deterioration of health under the stress of neurotoxins may also reduce production and activity of AChE^56^. Decline in the activity of AChE was visible as concentration dependent slowing of heart rate, slow motion and paralysis in the exposed hatchlings of all the species in the present study. Accumulation of acetyl choline due to inhibition of AChE has been related to altered nervous system function and paralysis^11^ and it became evident as a concentration and duration dependent alteration in feeding and swimming behavior and a gradual increase in paralytic larvae on exposure to TCS. Initially there was loss of spatial orientation and then the hatchlings became paralyzed with the increase in duration.

Hatchlings of all the species showed normal swimming till 5 days of exposure to all the concentrations of TCS, but after 5 days at 0.02 and 0.05 mg/L and after 10–14 days at 0.005 and 0.01 mg/L, the hatchlings lost equilibrium, turned towards one side, and then showed sporadic wriggling movements only. At 0.05 mg/L TCS, paralytic hatchlings were maximum in *L. rohita* (40%) and minimum in *C. carpio* (20%). Prolongation of altered swimming and feeding behavior of the exposed hatchlings throughout the recovery period in our study could have been due to the loss of cholinergic neurons under the stress of TCS as reported by Periera et al.^57^ and Li et al.^45^.

Increase in the activity of GST with the concentration of TCS in all the fish after 7 days exposure and 10 days of recovery period hints that conjugation reactions^58^ and synthesis of GST may have increased on these durations to get rid of TCS^59^. GSTs help in generating less toxic and more hydrophilic products of a xenobiotic. Olivera et al.^60^ and Martins et al.^61^ also observed higher GST activity in the TCS exposed embryos of zebrafish and *Pelophylax perezi*, respectively. Observation of Martinez-Paz^62^ that TCS increased expression of *GSTd3, GSTe1, GSTo1 and GSTt1* corroborates the concentration dependent increase in GST activity of the hatchlings till 7 days in our study. However, between 7–14 days of exposure, stress at the two higher concentrations of TCS (0.02 and 0.05 mg/L) seems to have overpowered the defense mechanisms and to negatively regulate GST level in *C. carpio*, *C. idella* and *L. rohita.* This is corroborated by Ku et al.^28^ and Kaur and Kaur^63^.

It looks like that TCS exposed hatchlings needed more energy for overcoming the stress of TCS even during the post exposure period as LDH activity declined over 14 days values but was still more than the respective 7 days values. Increase in the activity of LDH with the concentration of TCS in the present study gets support from Martins et al.^61^ and Jha and Mitra-Mazumder^64^. For production of energy, conversion of pyruvate to lactate is catalyzed by LDH in the anaerobic pathway and the activity of LDH has been widely used as an indicator of cellular injury and changes in energy production in stressed animals^23,65,66^. Significantly higher activity of both AST and ALT in the present study after 7 and 14 days of exposure may have been for breakdown of free amino acids for production of additional energy for maintenance of homeostasis and is supported by Kumar et al.^67^ and Fu et al.^68^. Observations of Liu et al.^69^ that TCS increased the level of MAPK and p53 in the hatchlings and liver of adult of zebrafish, also support the increased activity of these metabolic enzymes throughout the exposure in the present study. When the stress of TCS subsided during the recovery period, a decline was observed in the activity of AST and ALT in all the species.

Increase in TCA cycle intermediates i.e. fumarate, succinate, malate and citrate during exposure and their high levels during the recovery period in all the species hints towards persistent alteration of metabolism of exposed hatchlings. Significantly higher activity of LDH, AST and ALT in the exposed hatchlings also points towards the metabolic stress of all the sublethal concentrations of TCS in the present study. This is corroborated by the observations of Mossa et al.^70^ that TCA cycle intermediates (especially succinate) are released from mitochondria and accumulate in the extra cellular matrix under stress. Under normal conditions, accumulation of organic acids never occurs^71^ but higher energy demand under stress may enhance TCA processes, which may lead to build up of intermediates^72^. Stress induced inhibition of succinate dehydrogenase and alterations of the normal citric acid cycle to an alternative, partially inverted citric acid cycle have been suggested to be responsible for increase in the level of succinate in cells^73,74^. Higher levels of citrate and abnormal carbohydrate metabolism have been reported in stressed fish by Fu et al.^68,75^.

Higher star plot area of GSH, GSSG and GST during the initial 7 days of exposure indicates that these biomolecules helped in overcoming the oxidative stress of TCS. As the stress prolonged further, increase in AChE, AST and ALT plot areas shows cellular or neuronal damage. During the recovery period, an increase in the area of organic acids indicates predominance of metabolic stress during the post exposure period. Based on IBR analysis, GSH, GSSG, AChE, AST, ALT could be ascertained as biomarkers for the stress of sublethal concentrations of TCS. Our results are in agreement with Paul et al.^76^.

Stress of TCS increased with the duration of exposure as there was a marked increase in mortality between 7–14 days of exposure. Marked increase in paralytic larvae between 5–14 days at 0.02 and 0.05 mg/L and between 10–14 days at 0.005 and 0.01 mg/L indicates that the selected concentrations of TCS gradually affected cytoskeleton and development of muscles^30^. Deflated swim bladder, yolk sac and cardiac oedema and delayed yolk sac absorption seem to be responsible for increased mortality of exposed hatchlings with the duration of exposure. These abnormalities indicate that even the sublethal concentrations of TCS have teratogenic effect on fish. Low energy level as the hatchlings ate very less seems to be an additional factor for increasing rate of mortality in TCS stressed hatchlings. Detailed discussion for the TCS induced abnormalities can be found in our previous study, Dar et al.^77^.

## Conclusion

The most common visible stress signs of TCS (oedema and slow heart rate) were observed only in 1–2% hatchlings at 0.01 mg/L while the change over control in biomarkers (decline in protein, GSH, GSSG and AChE but an increase in GST, LDH, AST, ALT, MDA and organic acids) was significant even at 0.005 mg/L. This highlights the usefulness of a set of biomarker molecules for assessing the stress response of fishes in contaminated waters. Data suggest suitability of GSH, GSSG, AChE, AST and ALT as biomarkers for the toxicity of sublethal concentrations of TCS during exposure as well as the post exposure period. Maximum variations in *L. rohita* but minimum variation in *C. mrigala* highlight the need for more comparative studies before making generalizations about the environmentally safe concentrations of TCS in water.

## Material and methods

Triclosan (catalogue number 72779-25G-F, purity ≥ 97.0%, HPLC) was purchased from Sigma-Aldrich. Stock solution of TCS was prepared in acetone (1 mg/mL) while filtered, dechlorinated tap water was used for making various dilutions.

Fish eggs were procured from the Government fish seed farm, Rajasansi, Amritsar, immediately after spawning and transported to the laboratory in oxygenated bags. After washing with saline, 30 min post fertilization embryos were exposed to control (tap water), solvent control (acetone, 0.05 mL/L) and four sublethal concentrations of TCS (0.005, 0.01, 0.02, and 0.05 mg/L TCS) according to OECD guidelines 210^78^. These values were approximately 1/20, 1/10, 1/5 and 1/2 of the 96 h LC_50_ values for *C. idella* (0.116 mg/L), *L. rohita* (0.096 mg/L) and *C. mrigala* (0.131 mg/L) but 1/40, 1/20, 1/10 and 1/5 of 96 h LC_50_ value for *C. carpio* (0.315 mg/L). The selected concentrations are as per our previous study, Dar et al.^77^. Approximately 1200 embryos (30 embryos per liter) of each fish were exposed in quadruplicate at room temperature (27.00 ± 5.00) in plastic pools of 40 L capacity. Exposure of 14 days was followed by a recovery period of 10 days. The 24 h old hatchlings were fed with boiled egg yolk at the rate of 2% of body weight once every day and test water was changed one hour after feeding. Average temperature, pH, dissolved oxygen (DO), electrical conductivity (EC), total dissolved solids (TDS) and total alkalinity of the test water during the experiment were 25.00 ± 4.24 °C, 7.35 ± 0.64, 7.70 ± 1.27 mg/L, 0.63 ± 0.07 mS/cm, 280.50 ± 2.12 mg/L and 385.00 ± 42.43 mg/L, respectively. All the experimental protocols in this study were approved by the Institutional Animal Ethics Committee, Guru Nanak Dev University.

### Bioaccumulation

Official method of AOAC, 2007.01^79^ was used for preparing whole larval extract for bioaccumulation studies. Hatchlings (300 mg) were homogenized in 1% acetic acid in acetonitrile (1 mL), and then anhydrous magnesium sulfate (200 mg) and sodium acetate (50 mg) were added to the homogenate. After 2 min of shaking, it was centrifuged at 1500 g for 5 min. The supernatant (0.5 mL) was mixed with anhydrous MgSO_4_ (75 mg) and primary secondary amine (PSA) sorbent (25 mg), centrifuged for 5 min at 1500 g, filtered through 0.22 μ filter and stored at 4° C till further use.

For GC–MS (Shimadzu Asia Pacific Ltd, Kyoto, Japan, model-QP2010 Plus with helium as a carrier gas and DB-5 ms column) analysis, initial temperature of the column was 50 °C, it was increased to 125 °C by a step up increase of 25 °C/min and then to 300 °C by a step up increase of 10 °C/ min. This temperature was held for 15 min, temperature of the sample injector was set at 250 °C, injection mode was split, column flow rate was 1.70 mL/min and 8 µL sample was injected for analysis. The ion source and interface temperatures were set to 200 °C and 280 °C, respectively. Standard curve in the range of 0.1–20 µg was used for quantification of TCS. Content of TCS has been expressed as µg/g tissue.

### Biochemical parameters

Biochemical parameters were estimated with the help of Systronics dual beam spectrophotometer- Genesis 10 UV. Dried and weighed larvae were homogenized in respective cold buffers, the crushing tubes were kept in ice during homogenization. After centrifugation for 40 min at 10,000 g, 4 °C, the supernatant was used for biochemical estimation.

### Protein content (mg/g tissue)

Method of Lowry et al. ^80^ was used for protein estimation with bovine serum albumin (BSA) as a standard. Standard curve was used for measuring the protein content.

### Total glutathione (GSH and GSSG)

Glutathoine (U/mg protein) was estimated by the method of Griffith^81^ from 10% homogenate in 1% picric acid. The reaction mixture consisted of 0.70 mL of NADPH solution (0.3 mM), 0.10 mL of DTNB solution (6.3 mM), 0.20 mL of distilled water, 10 µL GR and 5 µL sample. For GSSG assay, 100 µL sample was mixed vigorously with 2 µL vinylpyridine and allowed to stand for 1 h. The procedure was same to that for GSH, only 10 µL GR was replaced with 20 µL GR. Absorbance was recorded at 412 nm. Standard curves were prepared for both reduced and oxidized glutathione and quantity has been presented as µM/ mg protein.

### Melondialdehyde (MDA)

The malondialdehyde (MDA-µM/mg protein) estimation (based on TBA activity) was carried out according to Utley et al.^82^ in 10% homogenate prepared in 1.15% KCl. The reaction mixture consisting of 0.5 mL homogenate, 3 mL TCA (20%) and 1 mL aqueous solution of TBA (0.6%) was heated on a boiling water bath for 40 min, after cooling, 4 mL of n-butyl alcohol were added. After vigorous mixing, the solution was centrifuged at 3000 rpm for 10 min. Absorbance of the coloured butanol phase was recorded at 535 nm.

Calculations:

$${\text{b}} = \frac{{\Delta {\text{A}} \times {\text{V}}}}{{\varepsilon \times {\text{d}} \times {\text{v}}}} \times {\text{dilution factor}}$$

where, $\varepsilon$ = Extinction co-efficient (1.56 × 10^5^ M^-1^ cm^-1^), d = Light path (path length), ΔA = Change in absorbance, V = Total volume, v = volume of sample.

### Acetylcholineesterase (AChE)

Activity of AChE was analyzed according to Ellman et al.^83^. Acetylthiocholine iodide (0.075 M) was used as a substrate and 5% homogenate was made in extraction/estimation buffer (0.1 M potassium phosphate buffer, pH 8.0). The reaction mixture (1.56 mL) contained extraction/estimation buffer (1.3 mL), substrate solution (0.01 mL), dithio-bisnitrobenzoic acid (0.05 mL) and crude enzyme solution (0.2 mL). Absorbance was measured at 412 nm, at 30 °C for 5 min (at 1 min intervals). Specific enzyme activity has been expressed as µM/min/mg protein.

Calculations:

$$\begin{aligned} {\text{R}} & = \frac{\Delta {\text{A}}}{1.36{ }\left( {10^{4} } \right)} \times \frac{1}{(400/13120){\text{C}}_{0} } \\ & = 5.74\left( {10^{ - 4} } \right)\frac{\Delta {\text{A}}}{{\text{C}}_{0}} \\ \end{aligned}$$

where, R = Rate of hydrolysis of substrate in moles per minute per gram of tissue, ΔA =  Change in absorbance, C_0_ = Concentration of tissue (w/v).

### Glutathione s-transferase (GST)

Ten percent homogenate was prepared in 0.1 M sodium phosphate buffer (pH 7.6) containing 1 mM phenylthiourea (PTU) for measuring GST activity according to Chein and Dauterman^84^. The reaction mixture (1 mL) contained 0.1 mL substrate solution (0.1 mL of 95% ethanolic 1-chloro-2,4-dinitrobenzene-CDNB), 0.1 mL of 50 mM GSH, 25 µL of crude enzyme solution and 0.1 M sodium phosphate buffer (pH 7.6) containing 0.1 mM PTU. Absorbance was recorded at 340 nm, at 25 °C. Specific enzyme activity (b) has been expressed as µM/min/mg protein by the formula:

$${\text{b}} = { }\frac{{{\Delta A} \times {\text{V}}}}{{\varepsilon \times {\text{d}} \times \Delta {\text{t}} \times {\text{v}}}} \times {\text{dilution factor}}$$

where $\varepsilon$ = Extinction co-efficient (9.6 mM^−1^ cm^−1^), d = Light path (path length), ∆t = Time interval, ∆A = Change in absorbance per minute, V = Total volume, v = Volume of sample.

### Lactate dehydrogenase (LDH)

Activity of LDH (U/min/mg protein) was estimated according to Vassault et al.^85^ in 10% homogenate prepared in 81.3 mM tris/203.3 mM NaCl buffer (pH 7.2). The reaction mixture contained 2.5 mL tris/NaCl/NADH (0.244 mM NADH in tris/NaCl buffer), 0.05 mL crude enzyme extract and 0.5 mL tris/NaCl/pyruvate (9.76 mM pyruvate in tris/NaCl buffer). Absorbance was recorded at 340 nm at 1 min intervals for 5 min. Specific activity of the enzyme (b) was expressed as U/min/mg protein by the formula:

$${\text{b}} = 9682 \times \frac{{\Delta {\text{A}}}}{{\Delta {\text{t}}}}$$

where, ∆A = Change in absorbance, ∆t = Time interval.

### Aspartate transaminase (AST) and alanine transaminase (ALT)

Method of Wilkinson et al.^86^ was used for estimation of the activities of AST and ALT. Homogenate (10%) was prepared in 1.0 M potassium phosphate buffer (pH 7.4). The reaction mixture contained 1.3 mL of 0.1 M phosphate buffer (pH 7.4), 0.2 mL of crude enzyme extract, 0.2 mL of NADH solution, 1.0 mL of 0.375 M L-aspartate, 0.1 mL of malate dehydrogenase (5 U/mL) and 0.2 mL α-ketoglutarate (0.1 M). Methodology for ALT was same except for replacement of L-aspartate (0.375 M) with L-alanine (0.75 M) and malate dehydrogenase (5 U/mL) with lactate dehydrogenase (5 U/mL). Absorbance was recorded at 340 nm. Rate of change in absorbance per minute was measured ${ }(\Delta {\text{A}}/{\min})$ and the transaminase activity was calculated as U/min/mg protein.

$${\text{Transaminase }}\;{\text{activity}} = \frac{{\Delta {\text{A}}/{\min} \times 1000}}{{6.22 \times { }10^{3} }} \times \frac{3000}{{0.2}} = \frac{{{\Delta A}}}{{\min}} \times 2400{ }\;{\text{U}}/{\text{L}}$$

### Organic acids

GC–MS was performed for estimation of organic acids (fumarate, succinate, malate and citrate) according to the modified method of Sharma et al.^87^. To 25 mg of dry tissue, 0.25 mL of HCl (0.5 N) and 0.25 mL of methanol were added and the mixture was kept on a shaker for 3 h. The supernatant was collected after centrifugation for 10 min at 12,000 rpm. It was mixed with 150 μL methanol and 50 μL of 50% sulfuric acid (H_2_SO_4_) and incubated overnight at 60 °C in a water bath. After cooling to 25 °C, it was mixed with 400 μL chloroform and 200 μL distilled water and vortexed for 1 min. The lower layer was used for estimation of organic acids.

GC–MS (Shimadzu Asia Pacific Ltd, Kyoto, Japan, model-QP2010 Plus) analysis was performed with helium as a carrier gas and DB-5 ms column. Initial column temperature (50 °C) was held for 1 min, increased by 25 °C/min to 125 °C and then by 10 °C/min to 300 °C (held for15 min). Temperature of the sample injector was 250 °C, mode of injection was split and flow of gas was 1.7 mL/min. The ion source temperature was 200 °C and the interface temperature was set at 280 °C with solvent cut time as 3 min and relative detector gain mode. Mass spectra of citrate, fumarate, malate, and succinate were recorded from 2 µL sample according to the National Institute of Standard and Technology (NIST08s) and Wiley 7 library. Standard curves were prepared for each organic acid and quantity has been presented as µg/g dry tissue weight.

### Statistical analysis

The data were subjected to one-way ANOVA, Tukey’s test and Students t-test for determining statistical significance (p < 0.05) and have been expressed as mean ± SD. Integrated biomarker responses (IBR) were evaluated and stars plots were drawn for the biochemical markers of the selected species according to method Beliaeff and Burgeot^88^ Guerlet et al.^89^.
